# Antibiotic efficacy and resistance patterns of urinary tract infection-causing bacteria in dogs and resistome of multidrug-resistant *Klebsiella pneumoniae* via whole genome sequencing in South Korea

**DOI:** 10.3389/fvets.2024.1455021

**Published:** 2024-11-13

**Authors:** Da-Eun Lee, Ji-Yeon Hyeon, Seok-Won Kang, Dong-Yeop Lee, Jung-Hyun Kim

**Affiliations:** ^1^Department of Veterinary Internal Medicine, College of Veterinary Medicine, Konkuk University, Seoul, Republic of Korea; ^2^Department of Infectious Disease, College of Veterinary Medicine, Konkuk University, Seoul, Republic of Korea

**Keywords:** antibiotic susceptibility test, dog, fosfomycin, *Klebsiella pneumoniae*, multidrug resistance, urinary tract infection

## Abstract

Bacterial urinary tract infections (UTIs) are prevalent in dogs and necessitate antibiotic intervention. However, the emergence of multidrug-resistant (MDR) bacteria poses significant challenges to antibiotic therapy. Although fosfomycin has been demonstrated to achieve and maintain high concentrations in urine, suggesting its potential for treating UTIs in dogs, its efficacy and the resistance profiles of urinary pathogens from canine UTIs remain elusive. Therefore, this study was conducted to investigate the antibiotic susceptibility of bacterial pathogens isolated from companion dogs with UTIs, with a particular focus on their susceptibility and resistance to fosfomycin. A total of 70 isolates from urine samples were analyzed, of which *Escherichia coli* (*n* = 18), *Proteus mirabilis* (*n* = 9), *Klebsiella pneumoniae* (*n* = 5), and *Staphylococcus pseudintermedius* (*n* = 5) were predominant. Resistance to erythromycin was most prevalent (94.59%), followed by clindamycin (91.89%) and ampicillin (78.37%), whereas the lowest resistance rate was observed for amikacin (5.40%). Resistance to fosfomycin was observed in 15 out of the 37 predominant isolates (40.54%), including all *K. pneumoniae* isolates (100%). All isolates, except 4 *E. coli* strains, were categorized as MDR (33 out of 37; 89.18%). The resistance rates for amoxicillin/clavulanic acid and trimethoprim-sulfamethoxazole, which are common first-line antibiotics for canine UTIs, were 48.64 and 56.75%, respectively. Whole-genome sequencing of *K. pneumoniae* isolates, which exhibited high resistance to fosfomycin, revealed multiple antibiotic resistance genes, with chromosomal *fosA* present in all isolates. Among the 27 dogs with recurrent infection included in this study, 2 were administered fosfomycin, resulting in clinical remission, as evidenced by negative urine culture tests. Overall, this study is the first to demonstrate the importance of assessing fosfomycin resistance profile for optimal treatment of canine UTIs, particularly in cases involving MDR strains.

## 1 Introduction

Bacterial urinary tract infections (UTIs) are prevalent in small veterinary practices, affecting approximately 14% of companion dogs during their lifetime (1). Antibiotics are pivotal in the treatment of UTIs, with selection dependent on the antibiotic susceptibility profiles of the uropathogens (2). However, empirical antibiotic therapy is frequently initiated in veterinary clinics during the interim period awaiting susceptibility test results (3). Amoxicillin is commonly selected as the initial treatment, followed by trimethoprim-sulfadiazine (3). Notably, the misuse and overuse of antibiotics, particularly in cases of empirical administration without prior antibiotic susceptibility testing, have contributed to the emergence of multidrug-resistant (MDR) bacteria in both human and veterinary medicine.

The increasing prevalence of MDR bacteria in both human and veterinary medicine has significantly limited the range of antibiotics available for the treatment of UTIs. Notably, fosfomycin, discovered in 1969, has emerged as a viable alternative antibiotic. Recently, it has become a common choice for primary treatment in humans, particularly for uncomplicated UTIs and infections caused by MDR bacteria (4–6).

Fosfomycin is eliminated via glomerular filtration and exhibits higher concentrations in urine than in plasma, which enhances its effectiveness against urinary pathogens (7–9). Moreover, fosfomycin is effective against MDR bacteria, such as methicillin-resistant *Staphylococcus aureus*, vancomycin-resistant *Enterococcus*, extended-spectrum *β*-lactamase (ESBL)-producing *Enterobacteriaceae*, such as *Klebsiella pneumoniae*, and carbapenemase-producing *Enterobacteriaceae* (10, 11). Previous studies involving canine models have demonstrated that fosfomycin achieves and maintains high concentrations in urine, suggesting its potential for treating UTIs in dogs (12, 13). However, the efficacy of fosfomycin and the resistance profiles of urinary pathogens from canine UTIs remain largely unexplored.

This study was undertaken to investigate the antibiotic susceptibility and resistance profiles of major bacterial species isolated from companion dogs with UTIs, with a specific focus on their susceptibility and resistance to fosfomycin. Furthermore, we sought to examine the presence of antibiotic resistance genes in *K. pneumoniae*, which exhibited high resistance to fosfomycin, using whole-genome sequencing (WGS).

## 2 Materials and methods

### 2.1 Sampling

Between September 2019 and September 2022, a total of 221 clinical samples were collected from various lesions in dogs at the Veterinary Medical Teaching Hospital of Konkuk University in Seoul, South Korea. These samples included 52 urine specimens and were obtained from diverse anatomical regions, such as the skin, eyes, pleuroperitoneal effusion, gastrointestinal tract, and urogenital areas. All samples were promptly transported to the NosVet Laboratory (Gyeonggi-do, South Korea) and analyzed within 3–4 h for the isolation of causative agents through antibiotic susceptibility testing. The study protocol was reviewed and approved by the Institutional Animal Care and Use Committee (KU24059). Owners provided written informed consent for their dogs to participate in the study.

### 2.2 Bacterial isolation and identification

A total of 420 bacterial isolates were obtained from clinical samples, with 70 isolates derived from 52 urine samples. For bacterial isolation from urine samples, 50 μL aliquots of the samples were inoculated onto 2 blood agar plates and incubated at 37°C overnight under aerobic and anaerobic conditions. For anaerobic cultivation, the blood plate was placed in an anaerobic jar with an anaerobic gas pack. Identification testing was performed if at least 1 colony was detected on the blood agar plate; the colonies were identified using matrix-assisted laser desorption/ionization time-of-flight mass spectrometry (ASTA, Gyeonggi-do, South Korea). Among the 70 isolates from urine samples (Supplementary Table 1), the 4 predominant bacterial species (*n* = 37), isolated from 27 dogs, included *Escherichia coli* (*n* = 18), *Proteus mirabilis* (*n* = 9), *K. pneumoniae* (*n* = 5), and *Staphylococcus pseudintermedius* (*n* = 5). These species were subjected to further analysis.

### 2.3 Canine patient characteristics

Detailed information about the 27 dogs included in this study is presented in Table 1. The median age of the dogs was 10 years, with an age range from 2 to 17 years. Clinical signs of UTIs were observed in 20 dogs (74.07%), and 12 dogs (44.44%) had a history of recurrent infections. Concurrent conditions potentially affecting UTIs were identified as follows: hyperadrenocorticism in 10 dogs (37.03%), chronic kidney disease in 4 dogs (14.81%), urolithiasis in 2 dogs (7.40%), and acute renal failure in 1 dog (3.70%). Conditions unrelated to UTIs are not listed.

**Table 1:** Canine patient characteristics, antibiotic resistance profiles, and treatment outcomes.

Patient no.	Age	Sex	Breed	UTIs history		UTIs related comorbidities	Uropathogens	Resistant antibiotics	Prescribed antibiotics	Treatment outcome
				Clinical signs	Recurrence					
1	9	SF	Poodle	Subclinical	No	None	*E. coli*	VEC, E, AMP, DA	AMC	Remission
2	9	SF	Dachshund	Subclinical	Yes	None	*E. coli*	CL, KZ, CEC, CAZ, CTX, CFM, CPD, VEC, CN, E, AZM, OFX, ENR, MAR, AMP, AMC, DA, TE, DO, SXT, ATM, FOS	NIT	Remission
3	13	SF	Shih tzu	Subclinical	No	HAC, CKD	*E. coli*	VEC, E, AMP, DA, SXT	DO, DA, AMC	Remission
4	11	SF	Mixed	Hematuria	No	HAC	*E. coli*	E, DA	AMC	Remission
5	13	CM	Shih tzu	Stranguria	No	HAC, Urolithiasis	*E. coli*	CL, KZ, CEC, CAZ, CTX, CFM, CPD, VEC, CN, E, AZM, OFX, ENR, MAR, AMP, AMC, DA, ATM	AMC, DO	Remission
6	13	SF	Poodle	Urine dribbling	Yes	HAC	*K. pneumoniae*	CL, KZ, CEC, CAZ, CTX, CFM, CPD, VEC, CN, E, AZM, OFX, ENR, MAR, AMP, AMC, DA, SXT, ATM, NIT, FOS	DO	*E. coli* growth
				Urine odor	Yes	HAC	*E. coli*	CL, KZ, CEC, CAZ, CTX, CFM, CPD, VEC, CN, E, AZM, OFX, ENR, MAR, AMP, AMC, DA, IPM, TE, DO, SXT, ATM	NIT	Remission
7	13	SF	Chihuahua	Urine discharge	No	None	*E. coli*	CL, KZ, CEC, CAZ, CTX, CFM, CPD, VEC, CN, E, OFX, ENR, MAR, AMP, AMC, DA, TE, DO, ATM	AMC, ENR	Remission
8	10	CM	Schnauzer	Urine odor	Yes	None	*E. coli*	CL, KZ, CEC, CAZ, CTX, CFM, CPD, VEC, CN, E, AZM, OFX, ENR, MAR, AMP, AMC, DA, ITE, SXT, ATM	AMC, ENR, NIT, FOS	Remission*
9	10	CM	Pomeranian	Subclinical	No	None	*E. coli*	CL, KZ, CEC, CAZ, CTX, CFM, CPD, VEC, CN, E, OFX, ENR, MAR, AMP, AMC, DA, IPM, TE, DO, SXT	NIT	Remission
10	18	SF	Shih tzu	Hematuria, stranguria, polyuria, pollakiuria	Yes	CKD	*E. coli*	E, AZM, DA	AMC	NA
11	9	SF	Poodle	Polyuria	No	HAC, CKD	*E. coli*	CL, KZ, CEC, CAZ, CTX, CFM, CPD, VEC, E, AMP, AMC, DA, TE, ATM	STX	Remission
12	14	SF	Maltese	Urinary incontinence	No	None	*E. coli*	E, DA	AMC	NA
13	18	CM	Shih tzu	Subclinical	No	HAC, CKD	*E. coli*	E, OFX, ENR, MAR, DA	NIT	Remission
							*S. pseudintermedius*	CL, KZ, CEC, CAZ, CTX, CFM, CPD, VEC, CN, E, AZM, OFX, ENR, MAR, AMP, AMC, DA, IPM, TE, DO, SXT, OX, FOS		
14	6	SF	Bichon Frise	Subclinical	Yes	None	*K. pneumoniae*	E, AZM, AMP, DA, FOS	AMC	*K. pneumoniae* regrowth
15	8	CM	Poodle	Subclinical	Yes	Urolithiasis	*K. pneumoniae*	CL, KZ, CEC, CFM, CPD, VEC, CN, E, AZM, OFX, ENR, MAR, AMP, AMC, DA, NIT, FOS	AMC, DO, AK	Remission
16	10	CM	Yorkshire Terrier	Urine odor, cloudy color	Yes	HAC	*K. pneumoniae*	CL, KZ, CEC, CAZ, CTX, CFM, CPD, VEC, CN, E, OFX, ENR, MAR, AMP, DA, TE, SXT, ATM, NIT, FOS	AMC	NA
17	6	SF	Bichon Frise	Hematuria, urine odor	Yes	HAC	*P. mirabilis*	KZ, CN, E, AZM, AMP, DA, TE, DO, SXT, NIT, FOS	AMC, ENR	Remission
							*K. pneumoniae*	CL, KZ, CEC, CAZ, CTX, CFM, CPD, VEC, E, AZM, OFX, ENR, MAR, AMP, AMC, DA, IPM, TE, DO, SXT, ATM, NIT, FOS	CFM, AMC, FOS	Remission*
18	5	SF	Poodle	Hematuria, urine odor	No	None	*P. mirabilis*	CL, KZ, CEC, CFM, CPD, VEC, E, AZM, OFX, ENR, MAR, AMP, AMC, DA, TE, DO, NIT, FOS	STX	Remission
19	6	SF	Poodle	Urinary incontinence	Yes	None	*P. mirabilis*	CL, KZ, CEC, CFM, CPD, VEC, E, AZM, OFX, ENR, MAR, AMP, AMC, DA, TE, DO, SXT, NIT, FOS	AMC	Remission
20	13	CM	Yorkshire Terrier	Polyuria, urine dribbling	No	HAC	*P. mirabilis*	AK, DA, TE, DO, NIT, FOS	AMC, ENR	Remission
21	2	SF	Bichon Frise	Hematuria	Yes	None	*P. mirabilis*	E, AZM, DA, TE, DO, NIT	AMC	Remission
22	12	SF	Poodle	Hematuria	Yes	HAC	*P. mirabilis*	CL, KZ, CEC, CTX, CPD, VEC, E, AZM, ENR, AMP, DA, TE, DO, SXT, NIT, FOS	AMC	Remission
							*S. pseudintermedius*	E, AMP, DA, TE, DO		
23	11	CM	Maltese	Stranguria, pollakiuria	No	None	*P. mirabilis*	E, ENR, TE, DO, SXT, NIT, FOS	AMC	Remission
24	12	SF	Mixed	Hematuria	Yes	None	*P. mirabilis*	CL, KZ, CEC, CTX, CFM, CPD, VEC, CN, E, AZM, OFX, ENR, MAR, AMP, AMC, DA, IPM, TE, DO, SXT, NIT	AMC	Remission
25	5	SF	Poodle	Polyuria	No	Acute renal failure	*S. pseudintermedius*	VEC, CN, E, AZM, AMP, TE, DO, SXT	AMC	Remission
26	8	CM	Malamute	Urine odor	No	HAC	*S. pseudintermedius*	CN, E, AMP, DA, TE, DO, SXT	CL	NA
27	17	SF	English Cocker Spaniel	Pollakiuria	No	CKD	*S. pseudintermedius*	CL, KZ, CEC, CAZ, CTX, CFM, CPD, VEC, AMP, AMC, IPM, TE, DO, SXT, OX	ENR	NA

UTIs, urinary tract infections; SF, spayed female, CM, castrated male, HAC; hyperadrenocorticism, CKD; chronic kidney disease; CL, cephalexin; KZ, cephazolin; CEC, cefaclor; CAZ, ceftazidime; CTX, cefotaxime; CFM, cefixime; CPD, cefpodoxime; VEC, cefovecin; CN, gentamicin; AK, amikacin; E, erythromycin; AZM, azithromycin; OFX, ofloxacin; ENR, enrofloxacin; MAR, marbofloxacin; AMP, ampicillin; AMC, amoxicillin/clavulanic acid; DA, clindamycin; IPM, imipenem; TE, tetracycline; DO, doxycycline; SXT, sulfamethoxazole/trimethoprim; ATM, aztreonam; NIT, nitrofurantoin; OX, oxacillin; FOS, Fosfomycin; NA, not assessed.

*Remission after fosfomycin administration.

### 2.4 Antibiotic susceptibility test

Susceptibility testing for 25 antibiotics was performed using the Kirby-Bauer disk diffusion method, following the interpretive criteria recommended by the Clinical and Laboratory Standards Institute (CLSI) for consensus interpretation (14).

Susceptibility to fosfomycin was assessed using the agar dilution method, adhering to the CSLI and European Committee on Antimicrobial Susceptibility Testing (EUCAST) guidelines (14, 15). Minimum inhibitory concentrations (MICs) were determined using Mueller Hinton agar supplemented with 25 mg/L glucose-6-phosphate (Sigma Aldrich Co., South Korea), with fosfomycin trometamol (Sigma Aldrich Co., South Korea) tested in 2-fold dilutions ranging from 1 mg/L to 1,024 mg/L. The experiments were performed in triplicate, and results were interpreted using CLSI and EUCAST breakpoints/MICs. For *Enterobacterales* and *Staphylococcus* spp., MIC results were interpreted according to EUCAST guidelines, whereas for *E. coli*, interpretations were based on CLSI standards owing to their specificity and higher reliability (16, 17).

### 2.5 Whole-genome sequencing

Among the 37 bacterial isolates analyzed in this study, *K. pneumoniae* isolates (*n* = 5), which exhibited high resistance to fosfomycin, were subjected to WGS. Genomic DNA was isolated from pure cultures of *K. pneumoniae* isolates using the MagNA Pure 96 DNA and Viral NA Small Volume Kit on the MagNA Pure 96 instrument (Roche Applied Sciences, Germany), following the manufacturer’s instructions. DNA concentration was determined using a Qubit BR dsDNA assay kit (Invitrogen, Carlsbad, CA), and DNA samples at a concentration of 0.2 ng/μL were used for library preparation using the Illumina Nextera XT DNA Library Prep Kit (Illumina, San Diego, CA), following the procedures described in our previous study (18). The library pool, comprising 500 μL of 10 pM libraries, was loaded into the MiniSeq High Output Reagent cartridge (300 cycles) (Illumina). Paired FASTQ files were generated via base-calling from the raw read data obtained from Illumina sequencing.

### 2.6 Whole-genome sequencing analysis

Raw reads were trimmed using Bbduk^1^ (quality score [Q] > 20; minimum length > 50). Subsequently, the trimmed reads were assembled *de novo* using SPAdes 3.15.5 (19) with default settings in Geneious Prime 10 software. Contigs with coverage less than 5× and sizes below 300 bases were eliminated from the assembly. MLST 2.0 (Multi-Locus Sequence Typing) was used to determine the sequence types of the isolates. The presence of acquired antimicrobial resistance genes and chromosomal mutations in the *gyrA*, *gyrB*, *parC*, and *parE* genes was assessed using ResFinder 4.1^2^, applying a threshold of 90% and a minimum length of 60% with the assembled contigs.

## 3 Results

### 3.1 Antimicrobial treatments and clinical outcomes

Antibiotics were prescribed based on antibiotic susceptibility test results for all dogs (Table 1). Empirically prescribed antibiotics were maintained if the isolates were sensitive and replaced if resistance was detected. Amoxicillin/clavulanic acid (AMC) was the most frequently prescribed antibiotic, administered to 20 of the 27 dogs following susceptibility confirmation. Among the 37 bacterial strains isolated, susceptibility testing revealed a resistance rate of 48.64% (18/37) to AMC. AMC alone resulted in UTI remission in 8 cases. However, for 2 out of the 27 dogs with recurrent infections, no viable antibiotic options were available based on susceptibility test results. Despite initial effectiveness, the empirical prescriptions did not prevent recurrent infections, depleting available oral antibiotic options. In these cases, fosfomycin was administered at 40 mg/kg orally every 8 h for 2 weeks. Subsequent remission was achieved, as indicated by negative urine culture results and improved clinical signs (Table 1).

### 3.2 Antimicrobial resistance

The antibiotic resistance profiles for the 37 isolates are presented in Table 2, and the MIC values for fosfomycin by species are presented in Table 3. The highest observed resistance was against erythromycin (94.59%), followed by clindamycin (91.89%) and ampicillin (78.37%), whereas the lowest resistance rate was observed for amikacin (5.40%) (Table 2). Resistance rates for AMC and trimethoprim-sulfamethoxazole—common initial antibiotics for canine UTIs—were notably high at 48.64 and 56.75%, respectively (Table 2). Resistance rates against third-generation cephalosporins ranged from 40.54 to 64.86%. Among the antibiotics used for UTIs in dogs, the lowest resistance rate (40.54%) was observed for ceftazidime and nitrofurantoin, whereas the highest resistance rate was observed for cefovecin (64.86%) (Table 2). All isolates, except 4 *E. coli* strains, were categorized as MDR (89.18%) (data not shown). Two *S*. *pseudintermedius* isolates were resistant to oxacillin and categorized as methicillin-resistant *S. pseudintermedius* (MRSP).

**Table 2:** Antibiotic resistance of isolates from samples obtained from dogs with UTIs (*n* = 37).

Strains	Antibiotics*																								
	CL	KZ	CEC	CAZ	CTX	CFM	CPD	VEC	CN	AK	E	AZM	OFX	ENR	MAR	AMP	AMC	DA	IPM	TE	DO	SXT	ATM	NIT	FOS
*E. coli_01*	S	S	S	S	S	S	S	R	S	S	R	S	S	S	S	R	S	R	S	S	S	S	S	S	S
*E. coli_02*	R	R	R	R	R	R	R	R	R	S	R	R	R	R	R	R	R	R	S	R	R	R	R	S	R
*E. coli_03*	S	S	S	S	S	S	S	R	S	S	R	I	S	S	S	R	S	R	S	S	S	R	S	S	S
*E. coli_04*	R	R	R	R	R	R	R	R	R	S	R	R	R	R	R	R	R	R	S	R	R	R	R	S	R
*E. coli_05*	S	S	S	S	S	S	S	S	S	S	R	I	S	S	S	S	S	R	S	S	S	S	S	S	S
*E. coli_06*	R	R	R	R	R	R	R	R	R	S	R	R	R	R	R	R	R	R	S	S	S	S	R	S	S
*E. coli_07*	R	R	R	R	R	R	R	R	R	S	R	R	R	R	R	R	R	R	R	R	R	R	R	I	S
*E. coli_08*	R	R	R	R	R	R	R	R	R	S	R	S	R	R	R	R	R	R	S	R	R	S	R	S	S
*E. coli_09*	I	R	S	S	S	S	S	S	S	S	R	S	S	S	S	R	I	R	S	R	I	S	S	S	S
*E. coli_10*	R	R	R	R	R	R	R	R	R	S	R	R	R	R	R	R	R	R	I	R	I	R	R	S	S
*E. coli_11*	R	R	R	R	R	R	R	R	R	S	R	I	R	R	R	R	R	R	R	R	R	R	I	S	S
*E. coli_12*	R	R	R	R	R	R	R	R	R	S	R	I	R	R	R	R	R	R	S	R	I	R	R	R	S
*E. coli_13*	I	S	S	S	S	S	S	S	S	S	R	S	S	S	S	S	S	R	S	S	S	S	S	S	S
*E. coli_14*	R	R	R	R	R	R	R	R	S	S	R	I	S	I	S	R	R	R	S	R	I	S	R	S	S
*E. coli_15*	S	S	S	S	S	S	S	S	S	S	R	R	S	S	S	S	S	R	S	S	S	S	S	S	S
*E. coli_16*	R	R	R	R	R	R	R	R	R	S	R	R	R	R	R	R	R	R	S	R	R	R	R	S	S
*E. coli_17*	I	I	S	S	S	S	S	S	S	S	R	I	S	S	S	I	S	R	S	S	S	S	S	S	S
*E. coli_18*	I	I	S	S	S	S	S	S	S	I	R	I	R	R	R	S	S	R	S	S	S	S	S	S	S
Resistance rate (%)	55.5	61.1	55.5	55.5	55.5	55.5	55.5	66.6	50	0	100	38.8	55.5	55.5	55.5	72.2	55.5	100	11.1	55.5	33.3	44.4	50	5.55	11.1
*P. mirabilis_01*	R	R	R	S	I	R	R	R	S	S	R	R	R	R	R	R	R	R	S	R	R	S	S	R	R
*P. mirabilis_02*	S	R	S	S	S	S	S	S	R	S	R	R	S	S	S	R	S	R	S	R	R	R	S	R	R
*P. mirabilis_03*	R	R	R	S	I	R	R	R	S	S	R	R	R	R	R	R	R	R	S	R	R	R	S	R	R
*P. mirabilis_04*	S	I	S	S	S	S	S	S	S	R	I	R	S	S	S	S	S	R	I	R	R	S	S	R	R
*P. mirabilis_05*	S	S	S	S	S	S	S	S	S	S	R	R	S	S	S	S	S	R	S	R	R	S	S	R	S
*P. mirabilis_06*	R	R	R	S	R	S	R	R	S	S	R	R	S	R	I	R	S	R	S	R	R	R	S	R	R
*P. mirabilis_07*	R	R	R	S	R	S	R	R	S	S	R	R	S	R	I	R	S	R	S	R	R	R	S	R	R
*P. mirabilis_08*	S	I	S	S	S	S	S	S	S	S	R	I	S	R	S	I	S	I	S	R	R	R	S	R	R
*P. mirabilis_09*	R	R	R	S	R	R	R	R	R	S	R	R	R	R	R	R	R	R	R	R	R	R	S	R	S
Resistance rate (%)	55.5	66.6	55.5	0	33.3	33.3	55.5	55.5	22.2	11.1	88.8	88.8	33.3	66.6	33.3	66.6	33.3	88.8	11.1	100	100	66.6	0	100	77.7
*K. pneumoniae_38*	S	S	S	S	S	S	S	S	S	S	R	R	S	S	S	R	S	R	S	S	S	S	S	R	R
*K. pneumoniae_42*	R	R	R	I	I	R	R	R	R	S	R	R	R	R	R	R	R	R	S	S	S	I	S	R	R
*K. pneumoniae_43*	R	R	R	R	R	R	R	R	R	S	R	R	R	R	R	R	R	R	S	S	S	R	R	R	R
*K. pneumoniae_44*	R	R	R	R	R	R	R	R	R	S	R	I	R	R	R	R	I	R	S	R	I	R	R	R	R
*K. pneumoniae_45*	R	R	R	R	R	R	R	R	R	R	R	R	R	R	R	R	R	R	I	R	R	R	R	R	R
Resistance rate (%)	80	80	80	60	60	80	80	80	80	20	100	80	80	80	80	100	60	100	0	40	20	60	60	100	100
*S. pseudintermedius_01*	S	S	S	S	S	S	S	R	R	S	R	R	S	I	S	R	S	I	S	R	R	R	S	S	S
*S. pseudintermedius_02*	S	S	S	S	S	S	S	S	R	S	R	S	S	S	S	R	S	R	S	R	R	R	S	S	S
*S. pseudintermedius_03*	R	R	R	R	R	R	R	R	S	S	S	S	S	S	S	R	R	S	R	R	R	R	S	S	S
*S. pseudintermedius_04*	S	S	S	S	S	S	S	S	I	S	R	S	S	I	I	R	S	R	S	R	R	S	S	S	S
*S. pseudintermedius_05*	R	R	R	R	R	R	R	R	R	S	R	R	R	R	R	R	R	R	R	R	R	R	S	S	R
Resistance rate (%)	40	40	40	40	40	40	40	40	60	0	80	40	20	20	20	100	40	60	40	100	100	80	0	0	20
Total (*n* = 37) Resistance rate (%)	56.7	62.1	56.7	40.5	48.6	51.3	56.7	64.8	48.6	5.4	94.5	56.7	48.6	56.7	48.6	78.3	48.6	91.8	13.5	70.2	56.7	56.7	32.4	40.5	40.5

CL, cephalexin; KZ, cephazolin; CEC, cefaclor; CAZ, ceftazidime; CTX, cefotaxime; CFM, cefixime; CPD, cefpodoxime; VEC, cefovecin; CN, gentamicin; AK, amikacin; E, erythromycin; AZM, azithromycin; OFX, ofloxacin; ENR, enrofloxacin; MAR, marbofloxacin; AMP, ampicillin, AMC, amoxicillin/clavulanic acid; DA, clindamycin; IPM, imipenem; TE, tetracycline; DO, doxycycline; SXT, sulfamethoxazole/trimethoprim, ATM, aztreonam; NIT, nitrofurantoin; FOS, fosfomycin; R, resistant; S, sensitive; I, intermediate.

**Table 3:** Fosfomycin MIC results for the 37 bacterial species analyzed in this study.

Bacterial species	No. of isolates with the following MIC (mg/L)											Resistance rates (%)	
	Total	1	2	4	8	16	32	64^b^	128	256^a^	>256	CLSI	EUCAST
*E. coli*	18	0	0	0	0	0	2	5	9	2	0	11.11	88.88
*P. mirabilis*	9	0	0	1	0	1	0	2	3	0	2	22.22	77.77
*K. pneumoniae*	5	0	0	0	0	0	0	0	0	0	5	100	100
*S. pseudintermedius*	5	4	0	0	0	0	0	0	1	0	0	0	20

MIC, Minimum inhibitory concentration; CLSI, Clinical and Laboratory Standards Institute; EUCAST, European Committee on Antimicrobial Susceptibility Testing. CLSI^a^ and EUCAST^b^ Fosfomycin breakpoints.

The antibiotics to which the isolates exhibited 100% resistance, categorized by species, are as follows: erythromycin and clindamycin for *E. coli*; tetracycline, doxycycline, and nitrofurantoin for *P. mirabilis*; erythromycin, ampicillin, clindamycin, and nitrofurantoin for *K. pneumoniae*; and ampicillin, tetracycline, and doxycycline for *S. pseudintermedius*.

Resistance to fosfomycin was observed in 15 out of the 37 isolates (40.54%) based on our criteria. Specifically, resistance was observed in 100% of *K. pneumoniae*, 77.77% of *P. mirabilis*, 20% of *S. pseudintermedius*, and 11.11% of *E. coli* isolates (Table 2). However, following CLSI and EUCAST breakpoints, the resistance rates for *E. coli* were 11.11 and 88.88%, respectively; the resistance rates for *P. mirabilis* were 22.22 and 77.77%, respectively; all *K. pneumoniae* isolates (100%) exhibited resistance to fosfomycin according to both criteria; and the resistance rates for *S. pseudintermedius* were 0 and 20%, respectively (Table 3).

All *K. pneumoniae* isolates exhibited high levels of resistance to fosfomycin, with MIC values of >256 mg/L (Table 3). One isolate (*K. pneumoniae*_45) demonstrated intermediate susceptibility to imipenem while showing resistance to all other 24 antibiotics tested; therefore, it was classified as extensively drug-resistant (Table 2).

### 3.3 Whole-genome sequencing analysis

The 5 *K. pneumoniae* isolates were subjected to WGS analysis to determine their sequence types (ST) and identify antibiotic resistance genes (Table 4). The isolates were classified as ST 307 (*n* = 2), ST 3927 (*n* = 1), ST 11 (*n* = 1), and ST 395 (*n* = 1) (Table 4).

**Table 4:** Phenotypic and genotypic antibiotic resistance of *K. pneumoniae* strains.

Strain	KP 38		KP 42		KP 43		KP 44		KP 45	
KP sequence type	ST3927		ST11		ST395		ST307		ST307	
	P	G	P	G	P	G	P	G	P	G
Aminoglycoside susceptibility										
Amikacin	S		S	*aac(3)-IV* *aadA2b* *aph(4)-Ia* *aac(6′)-Ib-cr*	S		S	*aac(3)-IIa* *aph(3″)-Ib* *aph(6)-Id*	R	*aac(3)-IIa* *aadA2* *aph(3′)-Ia* *rmtB* *aph(3″)-Ib* *aph(6)-Id* *aac(6′)-Ib-cr*
Gentamicin	S		R		R		R		R	
Beta-lactam susceptibility										
Ampicillin	R	*blaLEN16*	R	*blaOXA-1* *blaSHV-182* *blaDHA-1*	R	*blaCMY-2* *blaSHV-182*	R	*blaSHV-106* *blaTEM-1B* *blaCTX-M-15*	R	*blaOXA-1* *blaSHV-106* *blaTEM-1B* *blaCTX-M-15* *blaDHA-1*
Amoxycillin/ Clavulanic acid	S		R		R		I		R	
Carbapenem susceptibility										
Imipenem	S	*ompK36 p.A217S* *ompK36 p.N218H* *ompK37 p.I70M* *ompK37 p.I128M*	S	ompK37 p.I70M ompK37 p.I128M ompK37 p.N230G ompK36 p.A217S	S	*ompK36 p.A217S* *ompK37 p.I70M* *ompK37 p.I128M* *ompK37 p.N230G*	S	*ompK37 p.I70M* *ompK37 p.I128M* *ompK37 p.N230G*	I	*ompK37 p.I70M* *ompK37 p.I128M* *ompK37 p.N230G*
Cephalosporin susceptibility										
Cephalexin	S	*ompK36 p.N49S* *ompK36 p.L59V* *ompK36 p.L191Q* *ompK36 p.F198Y* *ompK36 p.Q227N* *ompK36 p.L229V* *ompK36 p.N304E*	R	*ompK36 p.N49S* *ompK36 p.L59V* *ompK36 p.G189T* *ompK36 p.F198Y* *ompK36 p.F207Y* *ompK36 p.T222L* *ompK36 p.D223G* *ompK36 p.E232R* *ompK36 p.N304E*	R	*ompK36 p.N49S* *ompK36 p.L59V* *ompK36 p.G189T* *ompK36 p.F198Y* *ompK36 p.F207Y* *ompK36 p.T222L* *ompK36 p.D223G* *ompK36 p.E232R* *ompK36 p.N304E*	R	*ompK36 p.N49S* *ompK36 p.L59V* *ompK36 p.T184P*	R	*ompK36 p.N49S* *ompK36 p.L59V* *ompK36 p.T184P*
Cephazolin	S		R		R		R		R	
Cefaclor	S		R		R		R		R	
Ceftazidime	S		I		R		R		R	
Cefotaxime	S		I		R		R		R	
Cefixime	S		R		R		R		R	
Cefpodoxime	S		R		R		R		R	
Cefovecin	S		R		R		R		R	
Fluoroquinolone susceptibility										
Ofloxacin	S	*acrR p.P161R* *acrR p.G164A* *acrR p.F172S* *acrR p.R173G* *acrR p.L195V* *acrR p.F197I* *acrR p.K201M*	R	*qnrB4* *acrR p.P161R* *acrR p.G164A* *acrR p.F172S* *acrR p.R173G* *acrR p.L195V* *acrR p.F197I* *acrR p.K201M* *gyrA p.S83I* *parC p.S80I*	R	*acrR p.P161R* *acrR p.G164A* *acrR p.F172S* *acrR p.R173G* *acrR p.L195V* *acrR p.F197I* *acrR p.K201M* *gyrA p.S83I* *parC p.S80I*	R	*qnrB1* *acrR p.P161R* *acrR p.G164A* *acrR p.F172S* *acrR p.R173G* *acrR p.L195V* *acrR p.F197I* *acrR p.K201M* *gyrA p.S83I* *parC p.S80I*	R	*qnrB1* *qnrB4* *acrR p.P161R* *acrR p.G164A* *acrR p.F172S* *acrR p.R173G* *acrR p.L195V* *acrR p.F197I* *acrR p.K201M* *gyrA p.S83I* *parC p.S80I*
Enrofloxacin	S		R		R		R		R	
Marbofloxacin	S		R		R		R		R	
Tetracycline susceptibility										
Tetracycline	S		S		S		R	*tet(A)*	R	*tet(A)* *tet(G)*
Doxycycline	S		S		S		I		R	
Sulfonamide/Trimethoprim susceptibility										
Sulfamethoxazole/Trimethoprim	S		I		R		R	*sul2* *dfrA14*	R	*sul2* *dfrA12* *dfrA14*
Macrolide susceptibility										
Azithromycin	R		R	*mph(A)*	R		I		R	
erythromycin	R		R		R		R		R	
Lincosamide susceptibility										
Clindamycin	R		R		R		R		R	
Nitrofuran susceptibility										
Nitrofurantoin	R		R		R		R		R	
Monobactam susceptibility										
Aztreonam	S		S		R		R		R	
Fosfomycin susceptibility										
Fosfomycin	R	*fosA*	R	*fosA*	R	*fosA*	R	*fosA*	R	*fosA*
Other susceptibility										
Phenicol	ND		ND	*catB3* *floR*	ND		ND		ND	*catB3*
Disinfectant	ND	*OqxA* *OqxB*	ND	*OqxA* *OqxB* *qacE*	ND	*OqxA* *OqxB*	ND	*OqxA* *OqxB*	ND	*OqxA* *OqxB* *qacE*

P, phenotype; G, genotype; R, resistant; S, sensitive; I, intermediate; ND, not determined.

The isolates harbored multiple antibiotic resistance genes. All isolates carried the *fosA* gene and exhibited chromosomal mutations in *acrR*, *ompK37*, *ompK36*, *parC*, and *gyrA* (Table 4). The 2 ST 307 isolates exhibited a higher number of antibiotic resistance genes than the other ST isolates. These isolates harbored genes conferring resistance to aminoglycoside, beta-lactam, tetracycline, sulfonamide/trimethoprim, fosfomycin, and disinfectants (Table 4). However, none of the isolates harbored genes for resistance to lincosamide, nitrofuran, or monobactam (Table 4).

ESBL genes were also confirmed through WGS, with 4 out of the 5 *K. pneumoniae* isolates testing positive for ESBLs. The *BlaSHV* gene was identified in all 4 samples, whereas *blaTEM* and *blaCTX-M* co-existed in the ST 307 isolates.

## 4 Discussion

In this study, we investigated the antibiotic resistance profiles of UTI-causing bacteria isolated from dogs, focusing on their resistance to fosfomycin, and evaluated the clinical application and treatment response in dogs. Fosfomycin is currently recommended for treating UTIs, particularly severe UTIs caused by MDR *Enterobacteriaceae* in humans (4). Common uropathogens, including MDR isolates, have been demonstrated to exhibit high susceptibility to fosfomycin (20–23). Sabharwal and Sharma et al. reported that isolates with high levels of antimicrobial resistance, including *E. coli* and *P. mirabilis,* exhibited the highest susceptibility to fosfomycin (6). Additionally, Sreenivasan et al. identified fosfomycin as the sole oral antibiotic with significant *in vitro* antimicrobial efficacy against *Enterobacteriaceae* isolates (24). Consequently, the renewed interest in fosfomycin has prompted an increase in the number of studies investigating its susceptibility and resistance (25).

Variations in susceptibility and resistance rates were observed upon comparing fosfomycin susceptibility test results based on CLSI and EUCAST criteria. According to CLSI breakpoints, the rates of *E. coli*, *P. mirabilis*, *K. pneumoniae*, and *S. pseudintermedius* resistance to fosfomycin were 11.11, 22.22, 100, and 0%, respectively. Conversely, the resistance rates were 88.88, 77.77, 100, and 20%, respectively, based on EUCAST breakpoints (Table 3). Similarly, in a previous study, susceptibility rates for *E. coli*, *Klebsiella* spp., and other *Enterobacterales* were 92.9, 92.1, and 100% based on CLSI breakpoints. Conversely, susceptibility rates of 85.7, 86.9, and 92.9%, respectively, were reported based on EUCAST breakpoints (26, 27). The discrepancies between these criteria complicate the interpretation of fosfomycin susceptibility and resistance results when relying solely on agar dilutions. Therefore, additional genetic testing is warranted to achieve more accurate results, and establishing standardized criteria to reduce the variability in breakpoints between the two institutions is necessary for further investigation into fosfomycin susceptibility.

Studies on the susceptibility of MRSP to fosfomycin are limited. DiCicco et al. (28) analyzed 31 MRSP strains from dogs and reported a 77% susceptibility rate to fosfomycin according to EUCAST criteria. In our study, 80% (4 of 5) of *S. pseudintermedius* strains were susceptible to fosfomycin according to EUCAST criteria. Among the 5 strains, 2 were identified as MRSP, 1 of which exhibited resistance to fosfomycin. Further research is required to explore the mechanisms of fosfomycin resistance in *S. pseudintermedius* despite its anticipated efficacy against MDR strains.

*Klebsiella pneumoniae* is globally recognized as a significant disease-causing pathogen (29). Given the increasing incidence of *K. pneumoniae* infections in humans worldwide, the potential for transmission and resistance gene transfer in veterinary medicine cannot be overlooked. In a 2009 study conducted in South Korea, the susceptibility rate of *K. pneumoniae* from human patients to fosfomycin was 95.2% (30). However, this rate declined to 61.9% in a study by Cho et al., conducted from 2011 to 2013, indicating an increase in resistance (31). In the present study, all 5 *K. pneumoniae* isolates exhibited high-level resistance to fosfomycin. WGS revealed that the *fosA* gene was present in the chromosomal DNA of all *K. pneumoniae* isolates (data not shown). In a previous study by Huang et al., 80% of *K. pneumoniae* human isolates exhibited resistance to fosfomycin, and chromosomal *fosA* was identified in 98.8% of cases (32). The *fosA* gene can be located on either the bacterial chromosome or on a plasmid, with chromosomal *fosA* being widely recognized as a major determinant of high-level resistance to fosfomycin in various gram-negative species. This chromosomal presence represents a key contributor to the observed high-level resistance (33, 34).

All dogs from which *K. pneumoniae* was isolated in our study had recurrent UTIs and were repeatedly exposed to multiple antibiotics. While the median duration of antibiotic treatment for all dogs was 2 months, those with *K. pneumoniae* infections received treatment for a longer duration of 3.5 months, indicating prolonged antibiotic use. Fosfomycin was prescribed to 2 dog patients owing to the lack of alternative oral antibiotic options, resulting in remission in both cases. Despite agar dilution results indicating resistance to fosfomycin in 1 dog, treatment efficacy was observed, suggesting a possible discrepancy between *in vitro* and *in vivo* efficacy.

This study had a few limitations. First, the small sample size of bacterial isolates, which were collected from a single center, restricted the generalizability of the findings. Additionally, the unequal distribution of bacterial strains included in the analysis posed challenges in accurately comparing their resistance to fosfomycin. Furthermore, differences in MIC breakpoints for fosfomycin using the agar dilution method resulted in inconsistent results, rendering precise interpretation challenging. Finally, owing to the lack of clinical data on fosfomycin in veterinary medicine, the discussion predominantly relied on comparisons with fosfomycin studies conducted in humans.

In veterinary medicine, the MIC breakpoint for fosfomycin susceptibility testing remains undefined. Although MIC results provide *in vitro* susceptibility data, actual responses *in vivo* may differ from expected outcomes. The prediction of *in vivo* results in dogs is further complicated by the limited understanding of fosfomycin pharmacodynamics and the absence of extensive clinical trials. However, in cases where alternative antibiotic treatments are limited, fosfomycin could be considered an effective alternative, particularly when susceptibility is corroborated by sufficient evidence from urine bacterial culture and quantitative susceptibility testing.

## 5 Conclusion

To our knowledge, this study is the first to emphasize the importance of evaluating resistance to antibiotics, including fosfomycin, for the effective management of canine UTIs, particularly in cases involving MDR pathogens. This study revealed high levels of antibiotic resistance among UTI pathogens in dogs and highlighted the efficacy of fosfomycin against certain species, such as *E. coli*, *P. mirabilis*, and *S. pseudintermedius*, especially against MDR isolates. However, *K. pneumoniae* exhibited the highest level of resistance to fosfomycin, harboring multiple antibiotic resistance genes. In conclusion, this study provides key insights into antibiotic resistance in canine UTIs and the potential role of fosfomycin in the treatment of these infections. While fosfomycin shows promise as a treatment option for certain UTI-causing species, the high resistance observed in *K. pneumoniae* isolates is concerning. Therefore, caution is advised against its indiscriminate use owing to the risk of escalating resistance and transmission between animals and humans.

## Data Availability

The original contributions presented in the study are included in the article/Supplementary material. Further inquiries can be directed to the corresponding author. Paired-end reads of *K. pneumoniae* isolates from this study have been deposited in the National Center for Biotechnology Information (NCBI) under the Bioproject accession number PRJNA956693.

## Footnotes

^1^https://sourceforge.net/projects/bbmap

^2^https://cge.food.dtu.dk/services/ResFinder-4.1/
