# Supplementary material for: Antibiotic efficacy and resistance patterns of urinary tract infection-causing bacteria in dogs and resistome of multidrug-resistant Klebsiella pneumoniae via whole genome sequencing in South Korea
Source: Front Vet Sci. 2024 Nov 13;11:1455021. doi: 10.3389/fvets.2024.1455021 (PMC11599855; doi:10.3389/fvets.2024.1455021)
Supplement: Supplementary file 1 [file Table_1.DOCX]

Supplementary Material

**Antibiotic efficacy and resistance patterns**

**of urinary tract infection-causing bacteria in dogs and**

**resistome of multidrug-resistant *Klebsiella pneumoniae***

**via whole genome sequencing in South Korea**

**Da-Eun Lee^1†^, Ji-Yeon Hyeon^2†^, Seok-Won Kang^2^, Dong-Yeop Lee^2^ and Jung-Hyun Kim^1*^**

*** Correspondence:** Jung-Hyun Kim: junghyun@konkuk.ac.kr

# Supplementary Table

**Supplementary Table 1.** Bacterial isolates collected from the urine samples (*n*=70).

| **Gram** | **Species** | **No. of isolates (%)** |
| --- | --- | --- |
| Negative | *Escherichia coli* | 18 (25.71) |
|  | *Proteus mirabilis* | 9 (12.85) |
|  | *Klebsiella pneumoniae* | 5 (7.14) |
|  | *Achromobacter xylosoxidans* | 3 (4.28) |
|  | *Proteus vulgaris* | 2 (2.85) |
|  | *Comamonas acidovorans* | 1 (1.42) |
|  | *Elizabethkingia miricola* | 1 (1.42) |
|  | *Enterobacter cloacae* | 1 (1.42) |
|  | *Pseudomonas aeruginosa* | 1 (1.42) |
|  | *Pseudomonas chlororaphis* | 1 (1.42) |
|  | *Pseudomonas koreensis* | 1 (1.42) |
|  | *Serratia marcescens* | 1 (1.42) |
| Positive | *Staphylococcus pseudintermedius* | 5 (7.14) |
|  | *Enterococcus faecalis* | 3 (4.28) |
|  | *Enterococcus faecium* | 2 (2.85) |
|  | *Enterococcus gallinarum* | 2 (2.85) |
|  | *Corynebacterium urealyticum* | 2 (2.85) |
|  | *Alicyclobacillus tengchongensis* | 1 (1.42) |
|  | *Bacillus infantis* | 1 (1.42) |
|  | *Micrococcus luteus* | 1 (1.42) |
|  | *Staphylococcus capitis* | 1 (1.42) |
|  | *Staphylococcus caprae* | 1 (1.42) |
|  | *Staphylococcus epidermidis* | 1 (1.42) |
|  | *Staphylococcus hominis* | 1 (1.42) |
|  | *Staphylococcus schleiferi* | 1 (1.42) |
|  | *Streptococcus canis* | 1 (1.42) |
|  | *Streptococcus equinus* | 1 (1.42) |
|  | *Streptococcus gallolyticus* | 1 (1.42) |
|  | *Streptococcus mitis* | 1 (1.42) |
| Total |  | 70 |
